# Orally Administrated *Lactiplantibacillus plantarum* BGAN8-Derived EPS-AN8 Ameliorates Cd Hazards in Rats

**DOI:** 10.3390/ijms24032845

**Published:** 2023-02-02

**Authors:** Emilija Brdarić, Dušanka Popović, Svetlana Soković Bajić, Dina Tucović, Jelena Mutić, Maja Čakić-Milošević, Slađana Đurđić, Maja Tolinački, Aleksandra Popov Aleksandrov, Nataša Golić, Ivana Mirkov, Milica Živković

**Affiliations:** 1 Group for Probiotics and Microbiota-Host Interaction, Laboratory for Molecular Microbiology, Institute of Molecular Genetics and Genetic Engineering, University of Belgrade, 11042 Belgrade, Serbia; 2 Immunotoxicology Group, Department of Ecology, Institute for Biological Research “Sinisa Stankovic”—National Institute of Republic of Serbia, University of Belgrade, 11062 Belgrade, Serbia; 3 Faculty of Chemistry, University of Belgrade, 11158 Belgrade, Serbia; 4 Institute of Zoology, University of Belgrade-Faculty of Biology, 11158 Belgrade, Serbia

**Keywords:** cadmium, exopolysaccharides, sequestration, inflammation, oxidative stress, microbiota

## Abstract

Cadmium (Cd) is a highly toxic metal that is distributed worldwide. Exposure to it is correlated with a vast number of diseases and organism malfunctions. Exopolysaccharides (EPS) derived from *Lactiplantibacillus plantarum* BGAN8, EPS-AN8, previously showed great potential for the in vitro protection of intestinal cells from this metal. Here, we investigated the potential of food supplemented with EPS-AN8 to protect rats from the hazardous effects of Cd exposure. After thirty days of exposure to lower (5 ppm) and higher (50 ppm)-Cd doses, the administration of EPS-AN8 led to decreased Cd content in the kidneys, liver, and blood compared to only Cd-treated groups, whereas the fecal Cd content was strongly enriched. In addition, EPS-AN8 reversed Cd-provoked effects on the most significant parameters of oxidative stress (MDA, CAT, GST, and GSH) and inflammation (IL-1β, TNF-α, and IFN-γ) in the duodenum. Moreover, micrographs of the duodenum were in line with these findings. As the gut microbiota has an important role in maintaining homeostasis, we used 16S rRNA amplicon sequencing and investigated the effects of Cd and EPS-AN8 on one part of the microbiota presented in the duodenum. Although Cd decreased the growth of lactobacilli and mostly favored the blooming of opportunistic pathogen bacteria, parallel intake of EPS-AN8 reversed those changes. Therefore, our results imply that EPS-AN8 might be extremely noteworthy in combatting this toxic environmental pollutant.

## 1. Introduction

Cadmium (Cd) is a hazardous, non-biodegradable metal that is persistent in the environment. Epidemiological studies reported that even low exposures to Cd are linked to a number of adverse health effects related to malfunctioning of the kidney, liver, lungs, and cardiovascular and reproductive systems [1]. The bioaccessibility of Cd is a result of mining, smelting, and industrial use. Furthermore, anthropogenic activities are also a significant threat. Despite public health efforts, for the general population, contaminated food and water are the main routes of Cd exposure, except for exposure from smoking or occupational activities [2,3]. Widespread contamination of the soil, atmosphere, and water leads to Cd being ingested by plants and aquatic organisms and easily entering the food chain. It was documented that leafy vegetables, cereals, shellfish, potatoes, legumes, nuts, stem/root vegetables, and contaminated water contributed the most to Cd intake [3,4]. Therefore, humans are constantly exposed to this environmental pollutant. Properties of this metal, such as a long half-life [5] and low rate of excretion [6], mark it as a dangerous widespread pollutant. Thus, orally administrated Cd has important and inevitable effects on health. The gastrointestinal tract is the main target for exerting toxicity. Besides tremendous disruption and leakage of the intestinal barrier [7,8,9], Cd affects the gut microbiota, which is defined as an important factor in maintaining health, and leads to lipopolysaccharide (LPS)-induced up-regulation of proinflammatory gene expression [10,11]. In humans, 7% of Cd is absorbed and transported by the blood to other organs [12]. Cd accumulates in multiple tissue types, but the majority of Cd is accumulated in the liver and kidneys; consequently, its burden on the body increases with age [13,14,15]. This leads to numerous chronic diseases of the kidneys, liver, and lungs, as well as cardiovascular diseases, reproductive dysfunction, and increased mortality [12,16,17].

Considering all these facts, in our previous study [18], we proposed a strategy for putative protection against Cd’s hazardous effects using bacterial exopolysaccharides (EPS) derived from lactic acid bacteria (LAB), which are naturally present in GIT and have GRAS (‘generally recognized as safe’) status. Exopolysaccharides are known to be strain-specific carbohydrate polymers that might be covalently bound to the surface forming a capsule, loosely bound to surface, or secreted to the cell environment [19]. Bacterial EPS are commonly used in the food industry to stabilize products, improve their rheology, and prevent syneresis [20]. Nonetheless, they have been recognized as potent immunomodulatory and antioxidative molecules [21,22], which could be used as a substitution for live bacteria to reduce health risks and/or reliance on variable bacterial metabolism [23,24,25]. Furthermore, EPS contribute to the ability of bacteria to adsorb metal ions to their surface [26]. It has been well documented that the adsorption of heavy metals by EPS is based on physicochemical interactions between metal cations and negatively charged acidic functional groups of EPS (e.g., carboxyl, acetate, hydroxyl, amine, phosphate, and sulfate), which might result in physical sorption, ion exchange, complexation, and/or precipitation [27,28,29]. Previously, we showed that EPS-AN8 derived from *Lactiplantibacillus plantarum* BGAN8 exhibits a high Cd-binding capacity in an aqueous solution and provides protection from Cd-mediated toxicity in intestinal epithelial Caco-2 cells [18].

In this paper, we have tested the ability of EPS-AN8 as a food supplement to alleviate the hazardous effect of orally administrated Cd in an in vivo animal model. Therefore, the aims of the present study were to examine the efficiency of EPS-AN8 as a putative tool to mitigate adverse effects of prolonged Cd intake (30 days) related to the deposition of Cd in organs, Cd-induced intestinal oxidative stress, and inflammation in Dark Agouti (DA) rats. Furthermore, we followed the putative protective effect on gut microbiota.

## 2. Results

### 2.1. General Considerations

The administration of Cd was achieved through CdCl_2_ in distilled water and lasted 30 days. Rats were given Cd in a lower (5 ppm (mg/L)) and higher (50 ppm (mg/L)) concentration. Additionally, EPS-AN8 was administrated through food at a concentration of 100 µg/mL. During the experiment, an increase in body mass was observed, but there were no changes between groups. The average daily intake of Cd was based on the calculated water consumption, and it was 0.72 ± 0.1 mg/kg for the 5 ppm Cd group, 7.2 ± 0.3 mg/kg for the 50 ppm Cd group, 0.83 ± 0.1 mg/kg for the EPS-AN8/5 ppm group Cd, and 7.3 ± 0.3 mg/kg for the EPS-AN8/50 ppm Cd group. There were no differences detected in food and water intake. There was no evidence of death.

### 2.2. EPS-AN8 Decreases Cd Deposition/Accumulation in Tissues and Increases in Feces

**Figure 1:**
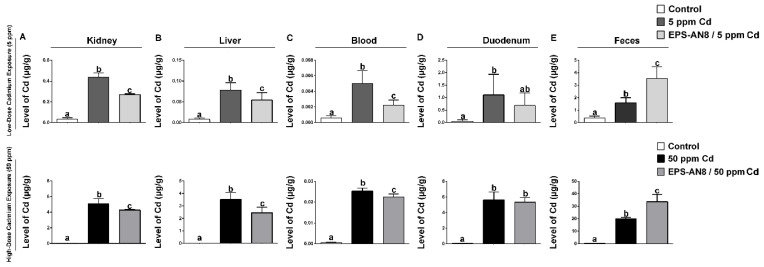
Effects of EPS-AN8 on Cd deposition in rats exposed to lower (5 ppm) and higher (50 ppm) dose of Cd in kidney (**A**), liver (**B**), blood (**C**), duodenum (**D**), and feces (**E**). Results are presented as mean ± standard deviation (SD). Values that do not share a common letter are significantly different (*p* < 0.05).

Consuming cadmium led to significantly higher accumulation in the organs of cadmium-treated groups than in the control group. The concentration of Cd was measured in the kidneys, liver, intestine, blood, duodenum, and fecal material (Figure 1). The organs most affected by this toxic metal were the kidneys, liver, and duodenum (Figure 1A,B,D). However, the level of Cd in groups who received EPS-AN8 combined with two doses (5 and 50 ppm) of Cd was significantly lower in the kidneys, liver, and blood compared to groups that administrated only Cd (Figure 1A–C). In the duodenum, there was no statistically significant difference between the levels of this harmful metal in groups treated with EPS-AN8 and Cd simultaneously and groups treated with just Cd (Figure 1D). Contrarily, the accumulation of Cd in feces was significantly higher in both groups that received Cd plus EPS-AN8 than in groups that simply received Cd, indicating that the body was excreting more cadmium (Figure 1E).

### 2.3. EPS-AN8 Reduces Histopathological Changes in Tissues

**Figure 2:**
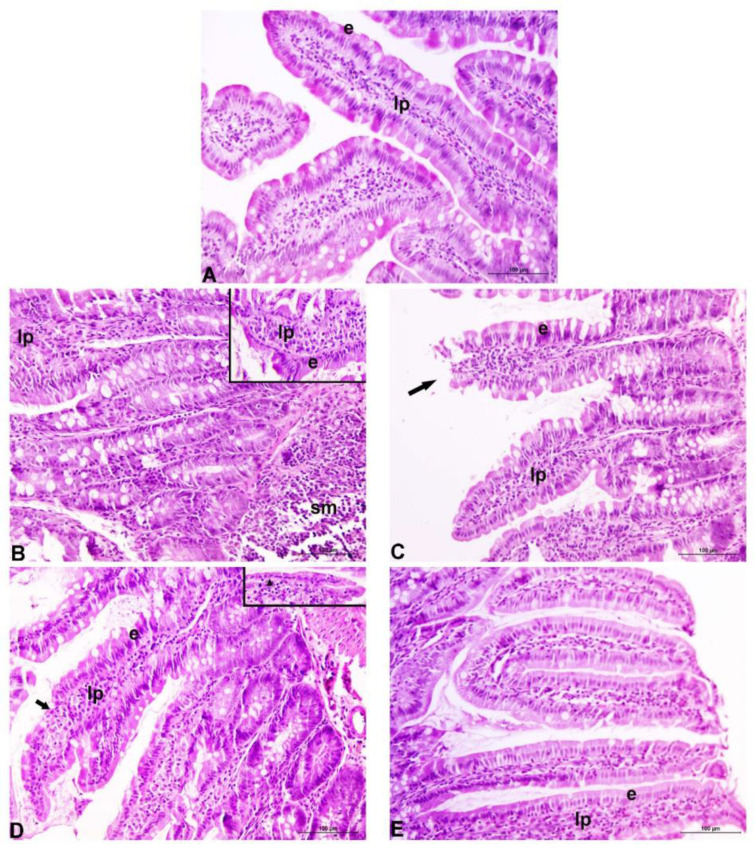
Representative photomicrographs of duodenum of rats exposed to different concentrations of Cd, with or without EPS-AN8. Normal appearance of duodenal mucosa in control rats (**A**). Thickened and shortened villi, increased leukocyte infiltration of the lamina propria and submucosa, and mild crypt hyperplasia after 5 ppm Cd treatment. Inset shows damaged surface epithelium along the villi (**B**). Generally better-preserved morphology of the villi despite some of them displaying apical damages after 50 ppm Cd (EPS-AN8 combined treatment. Arrow points to the damaged tip of the villus (**C**). Partial destruction of the duodenal epithelium (arrow: denuded lamina propria), crypt hyperplasia with frequent mitoses after 50 ppm treatment. Inset: flattening of the surface epithelium (arrowhead) (**D**). Relatively maintained gross integrity of the villi, increased content of intervillous material presumed to be secreted mucin (**E**). e: epithelium; lp: lamina propria; sm: submucosa; original magnification 20×.

Under microscopic examination, the duodenums taken from control rats showed normal histological structures (Figure 2A). Long slender villi were overlayed with well-organized columnar epithelium comprising enterocytes and goblet cells with evenly aligned nuclei. The lamina propria was thin with normal cellular content. Normal histology of the duodenal mucosa was affected by orally ingested Cd in a dose-dependent manner. In rats exposed to 5 ppm Cd (Figure 2B), villi were shortened and thickened, whereas crypts were characterized by mild hyperplasia. The epithelium was damaged in places and seemingly contained more mucin-producing cells. Increased leukocyte infiltration of the lamina propria and submucosa was noted. Most of these changes were similar or even more prominent in the 50 ppm Cd-treated group of rats (Figure 2D). Intake of EPS-AN8 visibly reduced these changes in both groups (Figure 2C, E). Notably, in the EPS-AN8/50 ppm Cd group, mucin-producing cells were less abundant, but an increase in the content of intervillous material, which is thought to be secreted mucin, was observed.

### 2.4. EPS-AN8 Mitigates Cd Induced Oxidative Stress in Duodenum

**Figure 3:**
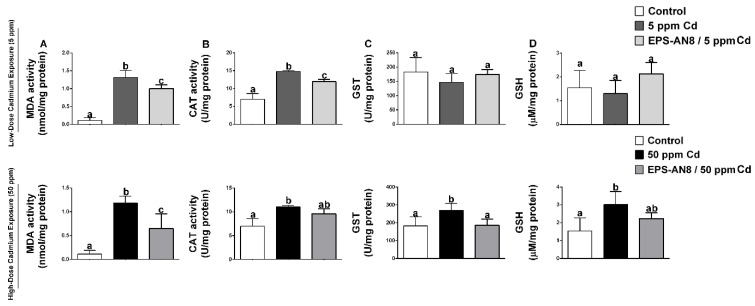
The protective effect of EPS-AN8 on Cd-induced oxidative stress are expressed via (**A**) MDA, (**B**) the acitivity of enzyme catalase, (**C**) the activity of glutathione-S-transferase, (**D**) the level of reduced glutathione. Results are presented as mean ± SD. Values that do not share a common letter are significantly different (*p* < 0.05).

The effects of the oral intake of Cd and EPS-AN8 on various parameters of oxidative stress in duodenum homogenates are presented in Figure 3. Cadmium administration at both doses significantly increased the amount of MDA in the duodenum (Figure 3A). Both EPS-AN8-treated groups had a lower level of MDA. Furthermore, the activity of catalase was higher in the Cd groups, but a decrease was observed in the groups given EPS-AN8 (Figure 3B). The activity of the enzyme GST, which catalyzes the conjugation of glutathione, was not affected by the lower dose of Cd. In contrast, the activity was significantly increased by 50 ppm Cd (Figure 3C). Oral intake of EPS-AN8 had a reversal effect on that alteration and maintained the measured values at the control level. Changes in the activity of GST might be considered to be adjustments to the increased level of GSH (Figure 3D). The lower dose of Cd did not cause any significant changes in GSH content, whereas the amount of GSH was increased in the duodenum of rats who received a higher dose of Cd. EPS-AN8 treatment decreased the level of GSH but without a statistical difference.

### 2.5. EPS-AN8 Alleviates Cadmium-Induced Cytokine Response in the Duodenum

**Figure 4:**
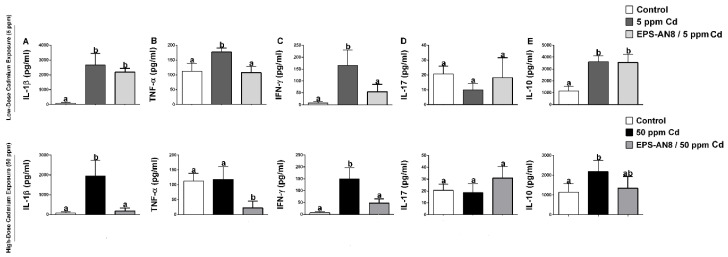
The ameliorating role of EPS-AN8 on Cd-induced cytokine production. It was measured production of (**A**) IL-1β, (**B**) TNF-α, (**C**) IFN-γ, (**D**) IL-17, (**E**) IL-10 Results are presented as mean ± SD. Values that do not share a common letter are significantly different (*p* < 0.05).

The cytokine profile is demonstrated in Figure 4. Oral intake of 5 ppm and 50 ppm Cd induced the overproduction of the potent pro-inflammatory cytokine IL-1β (Figure 4A). Although parallel intake of EPS-AN8 significantly protected against alterations in IL-1β content which was brought on by the higher dose of Cd, it did not appreciably reduce the overproduction of this cytokine that was caused by the dose of 5 ppm Cd. Further on, both groups of animals treated with only Cd had significantly upregulated production of TNF-α (except for 50 ppm Cd) and IFN-γ, whereas oral administration of EPS-AN8 significantly reduced this increase (Figure 4B,C). No changes were observed in the production of the inflammatory cytokine IL-17 in any of the examined groups compared to the control (Figure 4D). Immunoregulatory cytokine IL-10 was higher in Cd-treated groups compared to the control (Figure 4E). There were no differences between the group that took a lower dose of Cd and the group that was administrated EPS-AN8 and 5 ppm Cd. However, parallel intake of EPS-AN8 with a higher dose of Cd showed a trend in decreasing IL-10 production when compared to the 50 ppm Cd group, but those changes were not statistically significant.

### 2.6. EPS-AN8 Reverses Cd-Induced Changes in Gut Microbiota Composition

Alpha diversity as a parameter of biodiversity within the group was expressed via Shannon’s index, Simpson’s index, the Chao1 index, and the number of observed species (Figure S1). There were no changes between the control, Cd groups, and Cd groups treated with EPS-AN8 that were noticed according to the Shannon’s and Simpson’s indices (Figure S1A,B). More noticeable variations in microbial diversity and richness between groups were detected according to the Chao1 index and observed species (Figure S1C,D); however, the changes were not significant.

**Figure 5:**
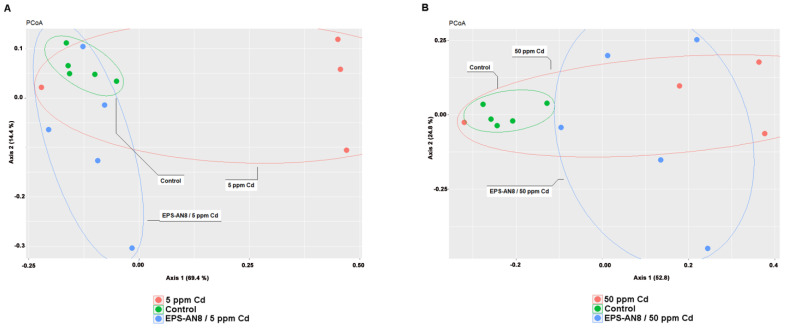
PCoA representation of microbial diversity comparison between control group and groups treated with lower dose of Cd (**A**); PCoA representation of comparison of microbial content between control group and groups treated with higher dose of Cd (**B**).

The beta diversity represents the composition of different microbial communities. PCoA, which is used for its visualization, revealed different clusterings of groups (Figure 5A,B) that were confirmed by Anosim (even though the difference was not statistically significant between the Cd groups and the EPS-treated Cd groups) (Table 1) and Adonis, as shown in Table 2.

**Figure 6:**
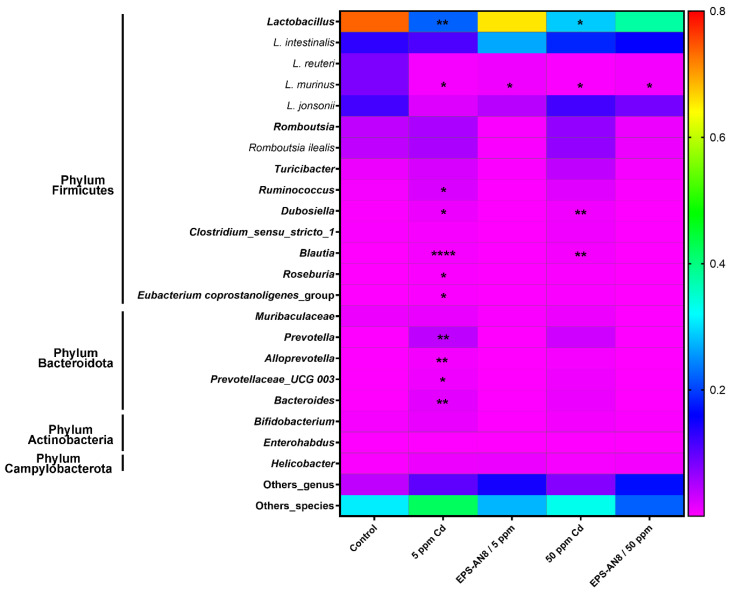
Relative abundance of genus and species in gut microbiota. Results are presented as median ± SD. Statistically significant differences at * *p* < 0.05, ** *p* < 0.01, and **** *p* < 0.0001.

In total, 18 OTUs had a relative abundance for genus and species higher than the selected threshold (0.001) in all the groups and were used for further analysis. The most changes in relative abundance were detected in phylum Firmicutes and phylum Bacteroidetes (Figure 6). *Lactobacillus* was the most abundant genus in all groups, but there was a significant decrease in its relative abundance in both pure Cd groups. In contrast, a lower concentration of Cd significantly up-regulated the relative abundance of five genera in the phylum Firmicutes (*Ruminococcus*, *Dubosiella*, *Blautia*, *Roseburia*, and *Eubacterium coprostanoligenes* groups) and four genera in the phylum Bacteroidota (*Prevotella*, *Alloprevotella*, *Prevotelaceae,* and *Bacteroides*), whereas a higher concentration significantly increased the relative abundance of two genera in the phylum Firmicutes (*Dubosiella* and *Blautia*). It is important to emphasize that the above-mentioned changes were not detected in Cd groups that were administrated EPS-AN8. In other words, the relative abundance of those genera did not differ from the control group. Variation in the relative abundance of species, which is reflected in a reduction in the abundance compared to the control, was noted only for *Lactobacillus murinus* (new classification: *Ligilactobacillus murinus* [30]).

**Figure 7:**
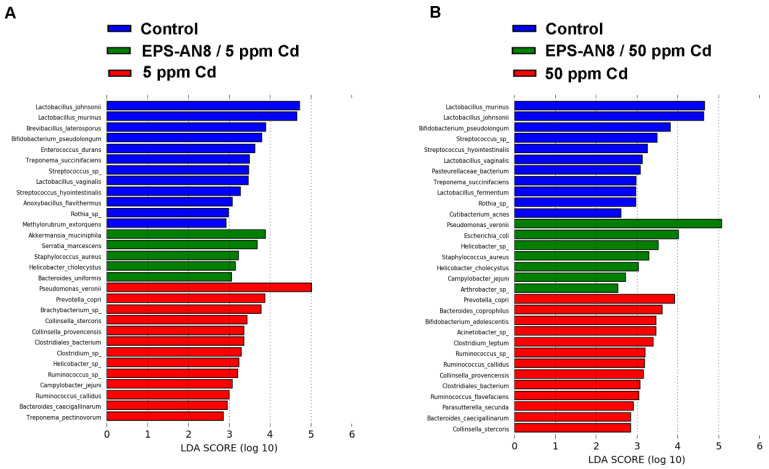
Graphical display of species between groups given by linear discriminant analysis effect size (LEfSe) of gut microbiota for lower (**A**) and higher (**B**) dose of Cd.

The linear discriminant analysis effect size (LEfSe) method revealed which genera and species were differentially represented among groups (Figure S2 and Figure 7). This method showed many different enriched genera between the control group and the EPS-AN8/5 ppm Cd and 5 ppm Cd groups, with *Akkermansia, Stenotrophomonas, Staphylococcus,* and *UCG_002* being the most abundant in the EPS-treated 5 ppm Cd group, and with *Pseudomonas, Prevotela, Bacteroides,* and the other differentially abundant genera in the 5 ppm Cd group having an LDA score above 2 (Figure S2A). Testing of the same groups indicated twelve species to be more abundant in the control group (*L. johnsonii* and *L. murinus* were specially enriched), five to be more abundant in the EPS-AN8/5 ppm Cd group (*Akkermansia muciniphila, Serratia marcescens, Staphylococcus aureus, Helicobacter cholecystus,* and *Bacteroides uniformis*), and thirteen to be more abundant in the 5 ppm Cd group (with *Pseudomonas veroni* and *Prevotella copri* as the most abundant) (Figure 7A). Comparison of the control, EPS-AN8/50 Cd ppm, and 50 ppm Cd groups demonstrated twelve genera to be more abundant in the control (*Streptococcus* as the most present), five to be more abundant in the EPS-AN8/50 ppm Cd group (*Pseudomonas, Escherichia Shigella, Campylobacter, Phascolarctobacterium*, and *Pseudarthrobacter*), and sixteen to be more abundant in the 50 ppm Cd group (*Rikenellaceae RC9 gut group, Anerobiospirillum, Elusimicrobioum,* and the others) (Supplement Figure S2B). Figure 7B presents differentially abundant species between control, EPS-AN8/50 Cd ppm, and 50 ppm Cd. There are eleven species more abundant in the control group (*L. johnsonii* and *L. murinus* having the highest LDA score), seven species more abundant for the EPS-AN8/50 ppm Cd group (*Pseudomonas veronii*, *Escherichia coli*, *Helicobacter* sp., *Staphylococcus aureus*, *Helicobacter cholecystus*, *Campylobcter jejuni*, and *Arthrobacter* sp.), and thirteen species more abundant for the 50 ppm Cd group (*Prevotela copri, Bacteroides coprophilus, Bifidobacterium adolescentis,* and the others).

## 3. Discussion

The increased number of Cd sources and knowledge of its harmfulness are attracting evermore attention [3]. Therefore, finding possible ways to limit, prevent, and remediate Cd’s hazardous effects has been the subject of a plethora of scientific studies [31,32]. The strategy of the application of microorganisms for the bio-removal of toxins and heavy metals has become favorable over the years. The reasons are due to the key characteristics of microorganisms as a treatment that is environmentally friendly, reasonably simple, and economically feasible [33]. Lactobacilli have stood out as one of the most promising beneficial biological sorbents in processes of reducing the bio-availability of toxins and heavy metals. The mixture of *L. rhamnosus* Rosel-11, *L. acidophilus* Rosel-52, and *Bifidobacterium longum* Rosell-175 mitigated the genotoxicity in vitro and in vivo by increasing the Cd level in feces and decreasing the Cd concentration in tissues and blood [34,35]. It was shown that the probiotic *L. plantarum* CCFM610 alleviates acute and chronic Cd toxicity via Cd sequestration, antioxidant effects, and protection of the intestinal barrier in mice [36,37,38]. The same probiotic strain promoted the growth and death arrest of Cd-exposed Nile tilapia fish (*Orechromis niloticius*) by decreased Cd accumulation, alleviation of oxidative stress, and normalizing of the hematobiochemical parameters [39]. Probiotics are defined as live microorganisms that, when administered in adequate amounts, confer a health benefit on the host (WHO-FAO, 2006). As such, they have been widely used to improve inherited microbial composition and to facilitate a return to eubiotic status. Nevertheless, probiotics might impair the return of indigenous microbiomes and provoke inflammation in compromised patients [25]. On the other hand, postbiotics, which are marked as bacterial-free metabolites secreted by probiotic strains, are recognized as a better and safer option. Therefore, recently, the substitution of probiotics with postbiotics has started to be a global trend in scientific research [25]. Some lactobacilli are able to produce homo- and hetero-polysaccharides with great structural diversity and divergent biological characteristics [40]. Based on their physio-chemical properties, some of them are highly potent in sequestering metal ions [41]. In our previous paper [18], we showed that, among other EPS-producing lactobacilli from our laboratory collection in aqueous solution, EPS-AN8 derived from *L. plantarum* BGAN8, which was originated from soft cow-milk cheese, has a remarkable capacity to bind Cd^2+^ions (more than 70%), a capacity which was favored by its qualitative composition. Additionally, in the same paper, we indicated that EPS-AN8 provides in vitro protection against Cd-mediated disruption of the intestinal barrier, as well as inflammation and oxidative stress. Therefore, we concluded that EPS-AN8 is a good candidate for further investigation.

To our knowledge, this is the first study with a unique approach that describes the importance and role of isolated EPS molecules in the protection of Cd harmfulness in vivo. Additionally, this study suggests that supplementing food with EPS might be an excellent strategy to restrict Cd spreading in organisms in highly polluted areas. In parallel, EPS-AN8 was administered to rats via rodent food. As mentioned above, Cd is accumulated in organisms and is a long-term health threat [14]. In this study, during lower and higher Cd exposure, EPS-AN8 was successful in significantly lowering Cd concentrations in the kidneys, liver, and blood, thereby limiting the spread of this metal in the body. Interestingly, in the duodenum, there was no significant change in the level of Cd deposition. The obtained results may be a consequence of the experimental setup. Namely, animals were given Cd-contaminated water and EPS-enriched food until the last day of the experiment. Therefore, it is reasonable that Cd was detected by ICP-MS, even though it is sequestrated by EPS-AN8 and forms a complex. It is known that Cd induces atony and decreases peristalsis, which causes constipation and a delay in excretion [42]. However, some studies suggest that EPS may improve the level of fecal moisture and wet weights of feces, thus preventing constipation [43]. Consumption of EPS-AN8 led to significant increased excretion of Cd through feces. This protective effect was also observed by probiotic strains [44], and this study demonstrates a similar effect of orally administrated EPS-AN8.

In addition, microscopically demonstrated histopathological alterations of duodenal mucosa in both Cd-treated groups indicate that Cd at applied doses and treatment durations damaged the intestinal barrier. Moreover, observations under the microscope supported the finding that EPS-AN8, when it was consumed together with Cd, protected from these harmful effects.

Two of the most important mechanisms of Cd toxicity are oxidative stress and inflammation [45]. These two processes are interconnected; one causes the manifestation of the other, and vice versa. Some studies have shown that lactobacilli might decrease parameters of oxidative stress such as MDA, CAT, SOD, and GSH in the liver and kidneys of mice acutely and chronically exposed to Cd [36]. As it was mentioned, GIT is the first target of orally ingested Cd; according to this, we have observed a range of oxidative stress and inflammatory status in the first part of the small intestine, the duodenum. Oral intake of EPS-AN8 significantly reversed the effects on Cd-mediated alterations of MDA, CAT (except for higher dose), GST, and GSH. Interestingly, a lower concentration of Cd did not significantly affect the GST and GSH. A low rate of Cd excretion causes terms of prolonged inflammation in organisms [9]. After 30 days of exposure, up-regulations of IL-1β, TNF-α, IFN-γ, and IL-10 were detected, whereas IL-17 was not affected. The administration of rodent choke supplemented with EPS-AN8 inhibited the changes. The reason for this amelioration effect of EPS-AN8 on Cd-mediated oxidative stress and inflammation might be based on the vast capacity for Cd^2+^ ion sequestration and incapability of Cd^2+^ ions to propagate a toxic effect, or it could be that EPS-AN8 directly affects the regulation of oxidative stress and inflammation because there is some evidence indicating that EPS molecules have a strong ability to reduce the inflammation. Such a case was with EPS derived from *L. paracasei* IJH-SONE68, which was isolated from fig leaf. These EPS inhibited the catalytic activity of hyaluronidase and the overexpression of ear IL-4 mRNA, which eventually led to anti-allergy and anti-inflammatory effects in picryl chloride-induced contact dermatitis [46]. Moreover, the same EPS prevented and ameliorated the inflammatory response of dextran-sulfate-sodium-induced ulcerative colitis in mice by decreasing mouse macrophage inflammatory protein 2 mRNA and stimulating the production of immunoregulatory cytokine IL-10 [47]. In addition, EPS which were isolated from *L. plantarum* YW11 reduced the production of the proinflammatory cytokines (TNFα, IL-1β, IL-6, IFN-γ, IL-12 and IL-18) and up-regulated IL-10, and this resulted in an amelioration of inflammatory bowel disease symptoms [48]. Our previous in vitro study [18] demonstrated that EPS-AN8 is capable of reducing NFκB-mediated inflammation 24 h after Cd enters the cells, supporting the idea that there may be more mechanisms by which EPS-AN8 protects against the detrimental effects of Cd in addition to the sequestration of ions.

It was evidenced that Cd alters gut microbiota [49,50], but the affected taxa were not consistent among studies. These discrepancies could be the result of differential gut microbiota between experimental animals, diverse vendors, environments, and different parts of gut microbiota used for the analysis. Furthermore, it is important to emphasize that different forms of Cd (cadmium-chloride, cadmium-glutathione, cadmium-citrate and cadmium-metallothionein), given by gavage, lead to distinct changes in the gut microbiota of the mice [31]. The gut includes the stomach, duodenum, ilium, and colon. The environment of those parts, from stomach to colon, is in the ascending queue of pH and anaerobic. In this study, we examined the microbiota found in the duodenum. Statistically insignificant changes for the alpha diversity of the tested groups were in concordance with a previous study by Richardson et al. (2018), in which the early stage response of rat gut microbiota to Cd exposure was followed. The Anosim test showed that variations between groups were higher than within groups, whereas Adonis indicated the percentage of different OTUs. The phyla most affected by Cd were Firmicutes and Bacteroidetes. Both the doses of Cd led to a reduction in the abundance of *Lactobacillus,* which confirmed the results from another study [51], and, interestingly, EPS-AN8 treatment prevented that change in both groups, which suggests its beneficial role in ameliorating Cd-induced perturbation of one part of the gut microbiota. Furthermore, both doses of Cd up-regulated the relative abundance of the genera *Dubosiella* and *Blautia*, which are Gram-positive anaerobic microbes. Although there is evidence of the beneficial role of representative species of *Dubosiella* in decreasing inflammation and oxidative stress, *Blautia* is usually increased in glucose metabolic disorders and metabolic syndromes in older adults and happens to be a marker of a prediabetic state and type 2 diabetes illness (T2D) [52]. It should be noted that, nowadays, the attribution of Cd, as an environmental pollutant, via its etiological role in the progression of T2D, has more frequently been the subject of various studies [53,54], and this finding may have an important role in describing its underlying putative mechanism. The administration of EPS-AN8 with both doses of Cd completely disabled these shifts. Moreover, a lower dose of Cd induced blooming of *Ruminococcus* and *Roseburia*; both genera are described as putative useful microbes for alleviating diverse pathological conditions [55,56]. Regarding the phylum Bacteroideta, the relative abundances of the genera *Prevotella, Alloprevotella,* and *Bacteroides* were enhanced in the duodenums of the rats exposed to a low dose of Cd. The tendency for the relative abundance of these genera to increase was also seen in the rats exposed to a higher dose of Cd. Representatives of *Prevotella* are commonly described in the literature as promotors of inflammation and inflammatory diseases such as ulcerative colitis and rheumatoid arthritis [57,58], whereas representatives of *Alloprevotela* have been found to be enriched in cancer tissue of oral squamous cell carcinoma [59] and up-regulated in fecal samples of patients suffering from chronic kidney disease (CKD) [54]. This was especially interesting because of the positive correlation between kidney diseases and the renal accumulation of Cd of people exposed chronically to this pollutant [60]. *Bacteroides* spp. act as opportunistic pathogens that are able to translocate from the gut to extraintestinal locations via extensive mucin degradation, which consequently leads to a compromised intestinal barrier and tissue damage [61]. However, these perturbations in the genera of phylum Bacteroides were reduced in the gut microbiota of animals whose food was supplemented with EPS-AN8. The obtained results indicate that the oral administration of EPS-AN8 shifted Cd-induced variation towards levels in the control group. Moreover, these changes in the relative abundance of OTUs were supported by LEfSe analysis, which revealed significant markers of diversity between the examined groups. Among others, *Prevotella copri*, which is known for its ability to dominate the intestinal microbiota of mice after colonization and increasing sensitivity for dextran sulfate sodium-induced colitis [62], was marked as a differential marker for a group of rats exposed to 5 ppm Cd. Furthermore, *Prevotella copri* was also detected as one of the differential markers for the Cd 50 ppm group, whereas *Pseudomonas veronni* appeared to be important in the same dose group but that was treated with EPS-AN8, which was also a marker for the 5 ppm Cd group. Interestingly, this was a non-pathogen species that could be a promising tool for sustainable wastewater biotreatments (removal, recovery, and biosensing) of Cd and copper (Cu) [63]. Thus, it is possible that this microenvironment with Cd that was presented in the lower range favors blooming of *P. veronni*. For the group that exposed to 5 ppm Cd but was treated with EPS-AN8, one marker was *Akkermansia muciniphila*. Intriguingly, *A. muciniphila* was identified as one of the most promising candidates for a next-generation probiotic which improves the gut barrier function and increases the production of mucins in the colon [64], but when orally ingested by mice that were acutely and chronically exposed to Cd, it failed to fulfil complete protection. The possible reasons are explained to be that it is highly sensitive to heavy metals and strongly influenced by the gut micro-environment [65]. Therefore, the presence of *A. muciniphila* is in concordance with our results of lower oxidative stress, inflammation, and tissue damage in the EPS-AN8-treated group.

**Figure 8:**
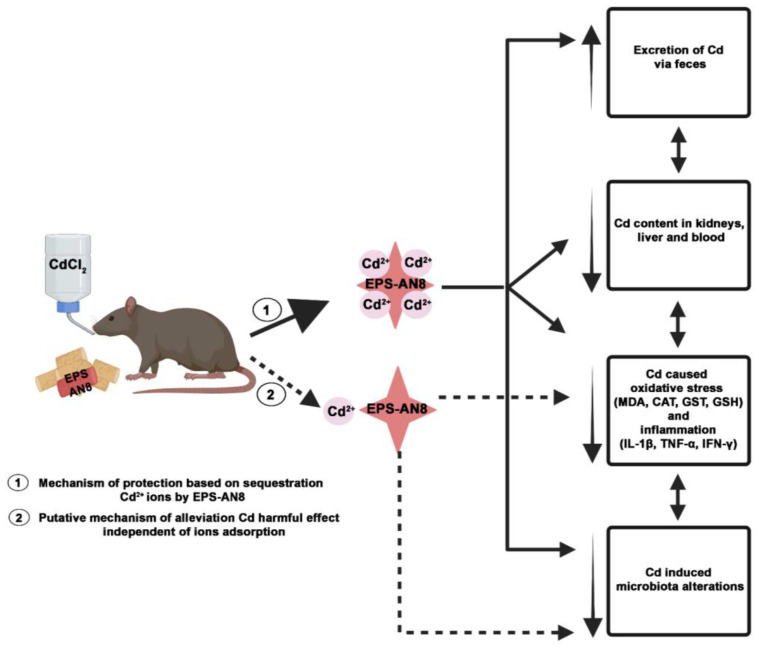
Proposed protective mechanisms of orally administrated EPS-AN8 against hazardous effects of Cd. Proposed mechanism marked with dashed lines remains to be confirmed. Created with BioRender.com.

Considering all these results, we proposed two mechanisms by which EPS-AN8 acts to combat orally ingested Cd (Figure 8). This paper evidenced changes after the parallel oral consumption of cadmium and EPS-AN8. Hence, we concluded that the extraordinary capacity of EPS-AN8 to bind with Cd^2+^ ions has an important role in protection. Consequently, EPS-AN8 strongly limited the spread of Cd in organisms and reduced the downstream effect on oxidative stress and inflammation. Thus, the first mechanism would be completely based on sequestration of Cd^2+^ ions by EPS-AN8. The second proposed mechanism assumed a direct effect of EPS-AN8′s role in recovery after Cd-induced damage on oxidative stress, inflammation, and microbiota. This mechanism is unbiased to Cd adsorption and may have a great impact on the protective role of EPS-AN8. However, to confirm the second assumption, further investigations need to be conducted.

## 4. Materials and Methods

### 4.1. Bacterial Strain, Media, and Growth Conditions

*Lactiplantibacillus plantarum* BGAN8 was grown at 30 °C in De Man Rogosa Sharpe medium (MRS (Merck, GmbH, Darmstadt, Germany)). MRS plates were prepared by adding 1.7% Agar (Torlak, Belgrade, Serbia).

### 4.2. Isolation and Purification of Exopolysaccharide

Exopolysaccharides produced by *Lactiplantibacillus plantarum* BGAN8 were extracted following the protocol by [66], with slight modifications described by [22]. Initially, for EPS isolation, 100 µL of overnight culture was spread on 500 MRS Agar plates. Isolation and purification were followed with five days of dialysis in Milli-Q water. The molecular mass cut-off of dialysis bags (Sigma-Aldrich, St. Louis, MO, USA) was 12–14 kDa. Finally, extracted and purified EPS were lyophilized (Alpha 1-4 LSC plus freeze dryer, Martin Christ, Germany).

### 4.3. Animals

All animal treatments and procedures were conducted in compliance with the Directive EU (86/609/EEC) on the care of animals used for experimental and other scientific purposes. Experiments were approved by the Veterinary Directorate, Ministry of Agriculture, Forestry and Water Management (No. 323-07-11824/2020-05). Dark Agouti (DA) 10–12-week-old male rats were conventionally housed and bred at the Institute for Biological Research ‘‘Sinisa Stankovic’’, University of Belgrade, under controlled conditions (12 h photoperiod, 21–24 °C temperature, and relative humidity of 60%).

### 4.4. Cadmium and EPS Treatment

Male DA rats were exposed to Cd through drinking water for a time period of 30 days at a concentration of 5 ppm (5 mg/L), which is experienced by people suffering from ‘itai-itai’ disease in Japan, whereas 50 ppm is proportionate to Cd found in people professionally exposed to this metal or living in highly polluted areas [67,68]. Cadmium was used in a form of cadmium chloride (CdCl_2_) and prepared in distilled water. Solutions of Cd and water were freshly prepared and changed twice a week. EPS-AN8 was ingested through rodent chow in a concentration 100 µg/mL, which corresponded to approximately 2 × 10^9^ CFU/mL. Rats were randomly divided into five major groups (control group, 5 ppm Cd group, 50 ppm Cd group, 5 ppm Cd plus EPS-AN8 group, and 50 ppm Cd plus EPS-AN8 group). Each group contained 4–5 animals. The control group received distilled water and rodent chow. There was one rat per cage. After a period of 30 days, animals were sacrificed by intraperitoneal injection of 15 mg/kg b.w. of Zoletil 100 (Virbac, Carros, France).

### 4.5. Cadmium Determination

All chemicals were of analytical grade and were supplied by Merck (Darmstadt, Germany). All glassware was soaked in 4 mol/L HNO_3_ for a minimum of 12 h and rinsed with ultra-pure water. Ultra-pure water was prepared by passing doubly de-ionized water from a Milli-Q system (Millipore Simplicity 185 System incorporating dual UV filters (185 and 254 nm) to remove carbon contamination).

Samples of kidneys, liver, intestine, blood, and feces were prepared for cadmium determination inside a clean laboratory without contamination. Microwave digestion was performed. Samples were transferred into PTFE cuvettes, and 7 mL of 65% HNO_3_ (*v*/*v*) and 1 mL 30% H_2_O_2_ (*v*/*v*) were added. Microwave digestion was performed in a Berghof microwave oven (Speedwave, Berghof, Germany). The digestion system was equipped with 12 PTFE vessels. Each sample was analyzed in duplicate, and each analysis consisted of three replicates. Digestion of samples was performed under the following program: heated for 10 min to 200 °C and held for 15 min at that temperature. After the cooling period, samples were quantitatively transferred into a volumetric flask (50 mL) and diluted with ultra-pure water.

The measurements of Cd in all samples were carried out in an ICP-MS (inductively coupled plasma mass spectrometry, iCAP Q, Thermo Scientific X series 2) that was equipped with flat pole collision cell technology (CCT), a micro-concentric nebulizer, platinum cones, and a peristaltic sample delivery pump and was running on quantitative analysis mode. The entire system was controlled with Qtegra Instrument Control Software. Instrument operating conditions are given in Table S1.

The stock solution containing 10 mg/L of Cd was used to prepare intermediate standard solutions for ICP-MS measurements. The internal standards used were ^45^Sc, ^115^In, and ^159^Tb. In order to check the accuracy and precision of instruments, the certified reference material DORM-2 (National Research Council of Canada, NRC-CNRC) was treated and analyzed in the same way as the samples. The results of the analyses were in accordance with the certified levels within a 95% confidence level.

### 4.6. Histology

The duodenums of rats were harvested and thoroughly rinsed in ice-cold physiological saline. A small piece of duodenum was fixed in 4% formaldehyde solution, pH 6.9, for 48 h and routinely processed for light microscopy (dehydrated in a rising series of ethanol solutions, cleared in xylene, and embedded in paraffin). Five-micrometer-thick tissue sections were mounted onto glass slides and stained with hematoxylin and eosin (H&E). Slides were evaluated for histomorphology changes and photographed using a Leica DMLB light microscope (Leica Microsystems, Wetzlar, Germany) that was equipped with a Leica DFC295 camera and LAS Core software.

### 4.7. Preparation of Duodenal Homogenates

Intestinal samples were homogenized by using an IKA T18 Basic Homogenizer (IKA Works Inc., Wilmington NC, USA) in ten volumes of sucrose buffer (10 mM Tris–HCl pH 7.6, 1 mM EDTA, 250 mM sucrose) containing 1 mM phenylmethylsulphonyl fluoride (PMSF) on ice. The following step was sonification (3 × 15 s on ice, at 30% of the maximum intensity amplitude) by a laboratory sonicator (Bandelin electronic, UW 2070, Berlin, Germany). Homogenates were then centrifugated (1000× *g* for 20 min, at 4 °C), and the collected supernatants were used for oxidative stress and cytokine measurements.

### 4.8. Lipid Peroxidation

The protocol described by [69] was used for the evaluation of lipid peroxidation. A mixture of intestinal homogenates and thiobarbituric reagent (0.375 % thiobarbituric acid, 15 % trichloroacetic acid, and Tris-HCl (pH 7.4)) was heated for 60 min 95 °C and centrifugated. The absorbance of the gained supernatant was measured at 535 nm using a spectrophotometer (Shimadtzu Corporation, Lakewood, CA, USA). The malondialdehyde (MDA) content was estimated by reference to a standard curve generated by known amounts of MDA and expressed as nmol of MDA/mg of protein.

Lowry assay was used for the determination of the protein concentration [70]. Briefly, intestinal homogenates were mixed with reagent C (2% Na_2_CO_3_ (Carlo Erba, Milano, Italy) in 0.1 M NaOH (LachNer, Neratovice, Czech Republic), 1% CuSO_4_×5H2O (Zorka, Šabac, Serbia), 2% KNaC_4_H_4_O_6_ × 4H_2_O (Alkaloid, Skopje, North Macedonia)), and 1 × Follin Ciocalteu’s phenol reagent. As a reference for the calculation of the protein concentration, the bovine serum albumin concentration was used (BSA, AppliChem, Darmstadt, Germany). Absorbance was measured at 670 nm using a spectrophotometer.

### 4.9. Determination of Glutathione-S-Transferase (GST) and Reduced Glutathione (GSH)

The activity of glutathione transferase (GST) was measured as the rate of the produced dinitrochlorobenzene (DNCB)–glutathione (GSH) complex catalyzed by this enzyme [71]. The GST activity directly corelates with the increase in the sample absorbance. The absorbance was monitored spectrophotometrically at a wavelength of 340 nm every 30 s for 180 s at 25 °C, and activity was expressed as units per milligram of protein (U/mg protein).

The glutathione (GSH) content was determined following the protocol described by [72]. Briefly, the supernatant fraction of intestinal homogenates was deproteinized (in 10% sulfosalicylic acid). Ellman’s reagent (5,5-dithio-bis-(2-nitrobenzoic acid)) in Tris–Cl (pH 8.9) and reduced glutathione were used as standard. Absorbance was measured at 412 nm, and data are expressed as µmol/mg.

### 4.10. Measurement of Catalase (CAT) Activity

CAT activity was estimated as the amount of H_2_O_2_ decomposition [73]. Intestinal homogenates were mixed with Tris-EDTA buffer (pH 8.8) and H_2_O_2_, and the absorbance change was measured spectrophotometrically at 240 nm, using a Shimadzu UV-1800 spectrophotometer, for 3 min (every 30 s) at 25 °C.

### 4.11. Cytokine Determination

Commercially available enzyme linked immunosorbent assays (ELISA) were used to determine cytokine concentrations in intestinal homogenates. IL-10, TNF-α, IL-1β (R&D Systems, Minneapolis, MN, USA), IL-17, and IFN-γ (eBioscience Inc., San Diego, CA, USA) were measured following the manufacturer’s instructions. The standard curve, which was constructed from a known amount of recombinant cytokines provided by ELISA sets, was used to calculate cytokine titer.

### 4.12. Duodenum DNA Extraction

Metagenomic DNA was extracted from the lumen of the duodenum using the commercially available kit ZR Tissue DNA MiniPrep™ Kit (Zymo Research Corp., Irvine, CA USA). The concentration of isolated DNA was measured on a BioSpec-nano spectrophotometer (Shimadzu, Columbia, MD, USA) and kept at −20 °C. All samples had the necessary concentration (≥12 ng/μL) and were sent to the Novogene Company (Cambridge, UK) in a final volume of 30 μL. The library was constructed, and the V3-V4 hypervariable region of 16S rRNA amplicon was sequenced using the Illumina NovaSeq paired-end platform. Quality control was performed at each step of the procedure. Each sample had a flattened refraction curve, which indicates a sufficing depth during sequencing.

### 4.13. Data Display and Statistical Analysis

FLASH (V1.2.7 http://ccb.jhu.edu/software/FLASH/, accessed on 3 June 2022) was used to merge paired-end reads. Quality filtering on the raw tags was performed under specific filtering conditions according to the Qiime (V1.7.0 http://qiime.org/scripts/split_libraries_fastq.html, accessed on 3 June 2022) quality-controlled process in order to obtain the high-quality clean tags. Species annotation of each representative sequence at each taxonomic rank was performed using Qiime (Version 1.7.0 http://qiime.org/scripts/assign_taxonomy.html, accessed on 3 June 2022) with the Mothur method against the SSUrRNA database of the SILVA Database. Normalized OTU data were used to perform Alpha and Beta diversity analysis. Alpha diversity was expressed through 4 indices, including the Shannon, Simpson, Observed-species, and Chao1 indices. Beta diversity was visualized using principal coordinates analysis (PCoA) that was calculated in QIIME (Version 1.7.0), displayed with R software (Version 1.4.1717), and expressed via Anosim,Adonis, and LefSe.

One-way analysis of variance (ANOVA) followed by Tukey’s tests was used for multiple comparison. Statistical analysis and the preparation of graphs were performed with GraphPad Prism 8 software. Different letters indicate significant differences between treatments (*p* < 0.05), except for the presentation of relative taxa abundance. Data are presented as mean values ± the standard deviations from different experiments, except for the relative abundances of OTUs, which are expressed as medians ± the standard deviations.

## 5. Conclusions

The findings from this study indicate the important role of bacterium-derived molecules, EPS, in the protection of Cd harmfulness in vivo. Orally ingested EPS-AN8 showed tremendous potential in alleviating the toxic effect of prolonged Cd exposure and might be a promising putative solution as a food supplement for populations that are in contaminated areas and are exposed to this dangerous metal. It still remains unclear if EPS-AN8 protection is based only on the vast capacity for Cd-binding ions and whether the improved conditions after using EPS-AN8 are merely a result of that or whether EPS-AN8 has the capability to act directly on Cd-induced damages. Therefore, future research should be continued in that direction.

## Figures and Tables

**Table 1:** Beta diversity of gut microbiota expressed through Anosim.

Anosim	R Value	*p* Value
Control: 5 ppm Cd	0.4625	0.0479
Control: EPS-AN8/5 ppm Cd	0.212	0.046
5 ppm Cd-: EPS-AN8/5 ppm Cd	0.3563	0.073
Control: 50 ppm Cd	0.5	0.0239
Control: EPS-AN8/50 ppm Cd	0.468	0.0085
50 ppm Cd: EPS-AN8/50 ppm Cd	0.075	0.285

**Table 2:** Beta diversity of gut microbiota expressed through Adonis.

Adonis	R^2^ Value	*p* Value
Control: 5 ppm Cd	0.3268	0.024
Control: EPS-AN8/5 ppm Cd	0.2426	0.035
5 ppm Cd-: EPS-AN8/5 ppm Cd	0.3088	0.052
Control: 5 ppm Cd	0.2909	0.032
Control: EPS-AN8/50 ppm Cd	0.3838	0.008
50 ppm Cd: EPS-AN8/50 ppm Cd	0.143	0.312

## Data Availability

The raw 16S rRNA gene sequences used in this study have been deposited in the European Nucleotide Archive (https://www.ebi.ac.uk/ena accessed on 1 December 2022). The study accession number is PRJEB56180, and the secondary accession number is ERP141097. The SILVA ribosomal RNA database is available at http://www.arb-silva.de/ (accessed on 3 June 2022). The script used for QIIME is available at http://qiime.org/scripts/assign_taxonomy.html (accessed on 3 June 2022), and the LEfSe platform can be found at http://huttenhower.sph.harvard.edu/galaxy/ (accessed on 29 June 2022).

## Supplementary Materials

The supporting information can be downloaded at: https://www.mdpi.com/article/10.3390/ijms24032845/s1.
