# Supplementary material for: Orally Administrated Lactiplantibacillus plantarum BGAN8-Derived EPS-AN8 Ameliorates Cd Hazards in Rats
Source: Int J Mol Sci. 2023 Feb 2;24(3):2845. doi: 10.3390/ijms24032845 (PMC9917968; doi:10.3390/ijms24032845)
Supplement: Supplementary file 1 [file ijms-24-02845-s001.zip › ijms-2111146-supplementary.pdf]

Supplementary material

Orally Administrated *Lactiplantibacillus plantarum* BGAN8-Derived EPS-AN8 Ameliorates Cd Hazards in Rats

Emilija Brdarić [1], Dušanka Popović [2], Svetlana Soković Bajić [1], Dina Tucović [2], Jelena Mutić [3], Maja Čakić-Milošević [4], Slađana Đurđić [3], Maja Tolinački [1], Aleksandra Popov Aleksandrov [2], Nataša Golić [1], Ivana Mirkov [2] and Milica Živković [1,*]

[1] Group for Probiotics and Microbiota-Host Interaction, Laboratory for Molecular Microbiology, Institute of Molecular Genetics and Genetic Engineering, University of Belgrade, 11042 Belgrade, Serbia

[2] Immunotoxicology Group, Department of Ecology, Institute for Biological Research "Sinisa Stankovic"—National Institute of Republic of Serbia, University of Belgrade, 11062 Belgrade, Serbia

[3] Faculty of Chemistry, University of Belgrade, 11158 Belgrade, Serbia

[4] Institute of Zoology, University of Belgrade-Faculty of Biology, 11158 Belgrade, Serbia

* Correspondence: milicanikolic@imgge.bg.ac.rs

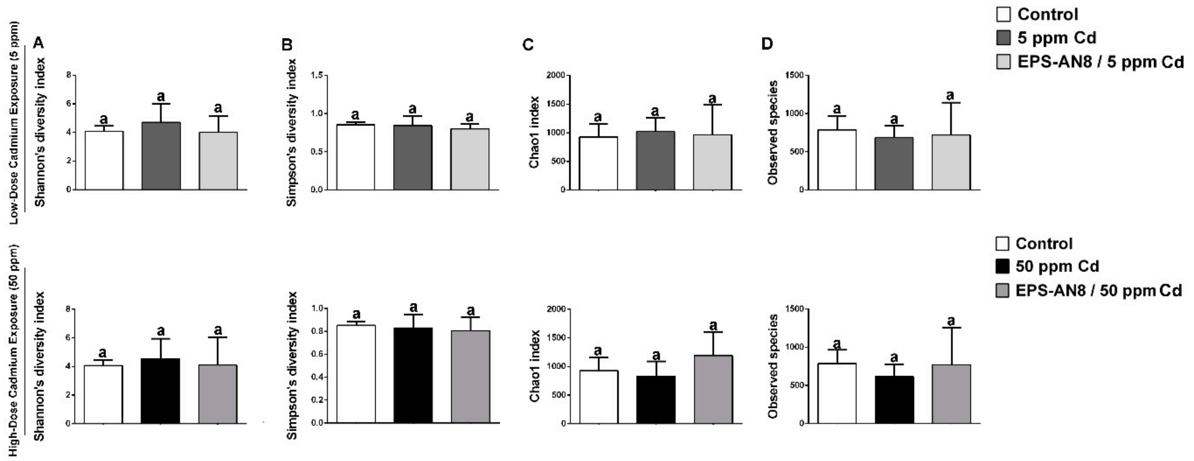

Figure S1. Alfa diversity of gut microbiota expressed via Shannon's diversity index (A), Simpson's diversity index (B), Chao1 index (C) and Observed species (D). Results are presented as mean ± SD. Values that do not share a common letter are significantly different ($p<0.05$).

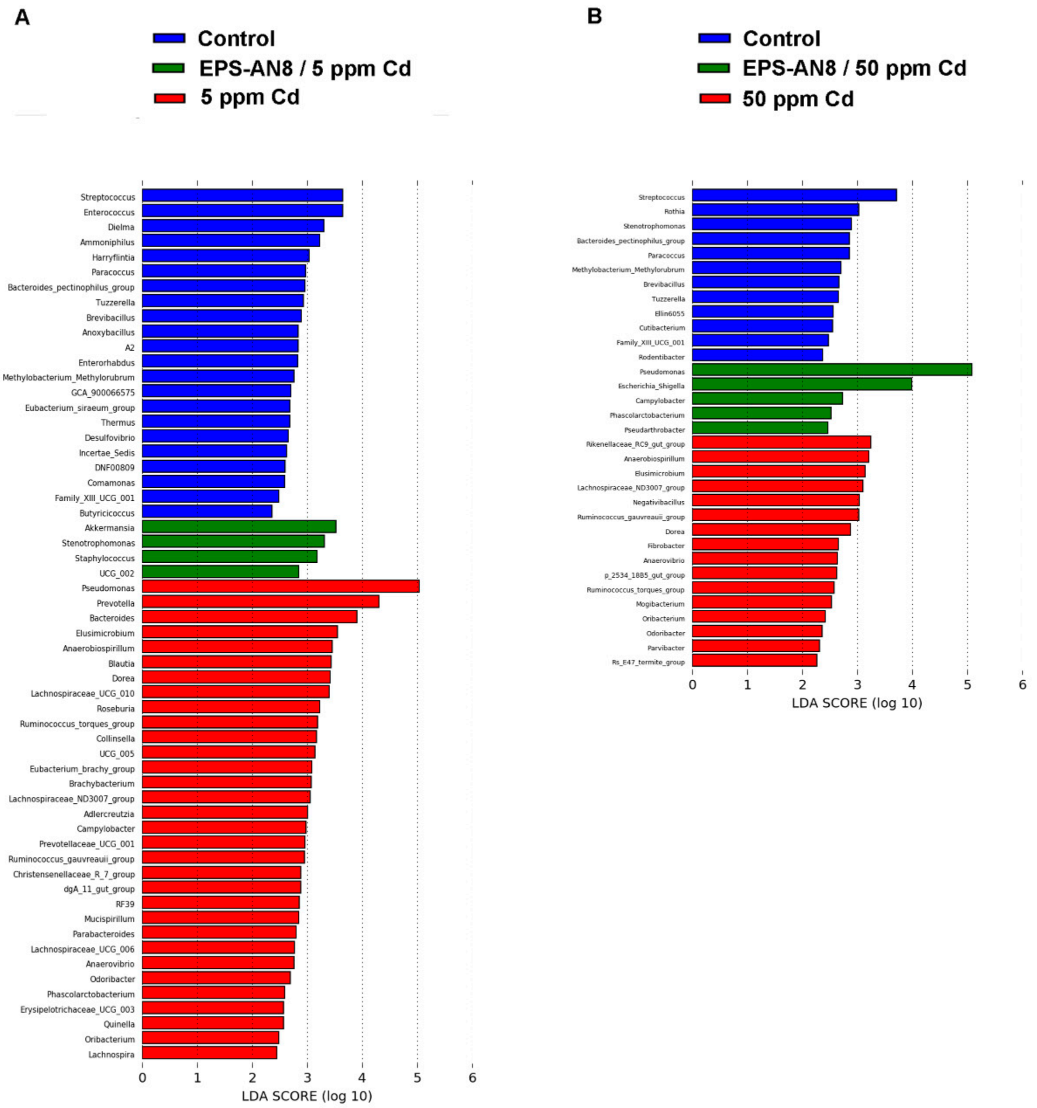

Figure S2. Representation of genera between groups given by Linear discriminant analysis effect size (LEfSe) method of gut microbiota for lower (A) and higher (B) dose of Cd.

Table S1. Instrument operating conditions of ICP-MS for Cd determination

Rf power (W)	1548
Gas flows (L/min)	13.9: 1.09; 0.8
Acquisition time	3 x 50 s
Points per peak	3
Dwell time (ns)	10
Detector mode	Pulse
Replicates	3
